# Multimorbidity and comorbidity in the Dutch population – data from general practices

**DOI:** 10.1186/1471-2458-12-715

**Published:** 2012-08-30

**Authors:** Sandra H van Oostrom, H Susan J Picavet, Boukje M van Gelder, Lidwien C Lemmens, Nancy Hoeymans, Christel E van Dijk, Robert A Verheij, François G Schellevis, Caroline A Baan

**Affiliations:** 1 Centre for Prevention and Health Services Research, National Institute for Public Health and the Environment, P.O. Box 1, Bilthoven 3720 BA, the Netherlands; 2 Centre for Public Health Forecasting, National Institute for Public Health and the Environment, P.O. Box 1, Bilthoven, 3720 BA, the Netherlands; 3 Department of General Practice, Netherlands Institute for Health Services Research, P.O. Box 1568, Utrecht, 3500 BN, the Netherlands; 4 Department of General Practice/EMGO Institute, VU University Medical Centre, Amsterdam, the Netherlands

**Keywords:** Multimorbidity, Comorbidity, Chronic disease, Epidemiology, Prevalence

## Abstract

**Background:**

Multimorbidity is increasingly recognized as a major public health challenge of modern societies. However, knowledge about the size of the population suffering from multimorbidity and the type of multimorbidity is scarce. The objective of this study was to present an overview of the prevalence of multimorbidity and comorbidity of chronic diseases in the Dutch population and to explore disease clustering and common comorbidities.

**Methods:**

We used 7 years data (2002–2008) of a large Dutch representative network of general practices (212,902 patients). Multimorbidity was defined as having two or more out of 29 chronic diseases. The prevalence of multimorbidity was calculated for the total population and by sex and age group. For 10 prevalent diseases among patients of 55 years and older (N = 52,014) logistic regressions analyses were used to study disease clustering and descriptive analyses to explore common comorbid diseases.

**Results:**

Multimorbidity of chronic diseases was found among 13% of the Dutch population and in 37% of those older than 55 years. Among patients over 55 years with a specific chronic disease more than two-thirds also had one or more other chronic diseases. Most disease pairs occurred more frequently than would be expected if diseases had been independent. Comorbidity was not limited to specific combinations of diseases; about 70% of those with a disease had one or more extra chronic diseases recorded which were not included in the top five of most common diseases.

**Conclusion:**

Multimorbidity is common at all ages though increasing with age, with over two-thirds of those with chronic diseases and aged 55 years and older being recorded with multimorbidity. Comorbidity encompassed many different combinations of chronic diseases. Given the ageing population, multimorbidity and its consequences should be taken into account in the organization of care in order to avoid fragmented care, in medical research and healthcare policy.

## Background

The presence of multiple coexistent chronic diseases is increasingly recognized as a major public health challenge of modern societies [1,2]. Challenges include optimalization of individuals’ health despite multimorbidity and the organisation and provision of health care for multimorbid patients. A first step in meeting these challenges is to provide insight into the size of the population suffering from multimorbidity, and the type of multimorbidity. Several studies on these figures are now available, showing high prevalences of multimorbidity [3-7]. Until now however, the distribution and combination of specific diseases received very little attention [8]. Health care needs of patients with multiple chronic conditions not only depend on the number but also, and maybe even more so, on the type of co-occurring diseases. With the ageing population and the accompanying rise in multimorbidity, the burden of chronically ill on health service capacity and costs is high [9] and is expected to rise in the future. Insight into combinations of diseases which often co-occur may assist in planning and improving (the organization of) health care services.

In this paper we analyze the prevalence of multimorbidity of chronic diseases by age and sex and the clustering of chronic diseases on the basis of electronic medical records in a representative sample of Dutch general practices. For a selection of 10 specific diseases we also present a detailed picture of the comorbidity, i.e. the type and prevalence of additional diseases among that specific disease.

## Methods

### Study design & participants

We used a longitudinal dataset of electronic medical records of over 350,000 patients in a representative sample of 92 general practices that participate in the Netherlands Information Network of General Practice (LINH), covering circa 2% of the total Dutch population [10]. General practices within this network are evenly distributed across the Netherlands. The listed population and the general practices are representative for the Dutch population and the Dutch general practitioners, respectively. In the Netherlands, all individuals are obligatory listed in a general practice, with exception of a small, very old part of the Dutch population in nursing homes. Therefore, the listed population can be used as the denominator in population based epidemiological studies. The prevalence of multimorbidity was analysed using LINH data from 2002 to 2008. LINH is registered with the Dutch Data Protection Authority; data are handled according to the data protection guidelines of the authority. According to Dutch legislation, studies using this kind of observational data do not require medical ethical approval.

### Dataset

LINH includes routinely recorded data on consultations, including drug prescriptions and referrals. Diagnostic codes were recorded with consultations, drug prescriptions, and referrals, according to the International Classification of Primary Care (ICPC) [11]. Episodes of care were constructed and included all patients contacts and drug prescriptions pertaining to a specific health problem [12]. Thus two consultations for the same health problem are grouped into one episode of care. Consider, for instance, a patient who visits the general practitioner with a chronic cough, and a few months later the same patient is diagnosed with COPD. Most likely, both diagnoses refer to the same health problem and to avoid double counting the two diagnoses were grouped into one episode of care named COPD. Another example is a patient with symptoms of breathlessness and a diagnosis of heart failure a few weeks later, these health problems were grouped into one episode of heart failure. These episodes were constructed by EPICON, an algorithm to group ICPC-coded contact records from electronic medical records in general practice into episodes of care [13,14].

For prevalence calculations the data from general practices should meet standards for completeness, i.e. for each general practice the percentage of valid ICPC codes should be 60% or more and the registration of morbidity and prescriptions must have occurred continuously over each year [10].

### Multimorbidity and comobidity

Multimorbidity is defined as the co-occurrence of two or more chronic diseases within one person in a specific period of time [15]. Comorbidity refers to the presence of at least one extra chronic disease along with a chronic disease of interest [16]. Chronic diseases are defined as irreversible conditions without complete recovery or relatively long-lasting conditions. Diseases were selected based on high prevalence and a chronic and severe character from a standard list of chronic conditions for primary care [17]. This resulted in 29 chronic diseases, presented with ICPC codes in Table1[17]. Comorbidity was explored for 10 chronic diseases which are prevalent among older patients: diabetes mellitus, coronary heart disease, osteoarthritis, chronic obstructive pulmonary disease (COPD), chronic neck- or back disorders, cancer, stroke, depression, heart failure, and anxiety disorders. 

**Table 1:** Selection of 29 chronic diseases with ICPC-1 codes

**Chronic disease**	**ICPC-1 code**
Tuberculosis	A70
HIV/AIDS	B90
Cancer	A79, B72, B73, D74, D75, D77, L71, N74, R84, R85, S77, T71, U75, U76, U77, W72, X75, X76, X77, Y77, Y78
Gastric or duodenal ulcer	D85, D86
Chronic enteritis/colitis ulcerosa	D94
Visual disorder	F83, F84, F92, F93, F94
Hearing disorder	H84, H85
Congenital cardiovascular anomaly	K73
Coronary heart disease	K74, K75, K76
Heart failure	K77
Stroke (including TIA)	K89, K90
Chronic back or neck disorder	L83, L84, L85, L86
Rheumatoid arthritis	L88
Osteoarthritis	L89, L90, L91
Osteoporosis	L95
Congenital neurological anomaly	N85
Multiple sclerosis	N86
Parkinson’s disease	N87
Epilepsy	N88
Chronic alcohol abuse	P15
Dementia	P70
Schizophrenia	P72
Anxiety disorder, neurosis, PTSS	P74, P79
Depressive disorder	P76
Mental retardation	P85
Chronic obstructive pulmonary disease	R91, R95
Asthma	R96
Anorexia	T06
Diabetes mellitus	T90

The selection of chronic diseases is based on ‘Defining chronic conditions for primary care using ICPC-2: supplementary data'.[17] ICPC-2 codes are encoded into ICPC-1 codes.

### Prevalence

Given that diagnoses are recorded based on patient contacts, the prevalence of some chronic diseases is underestimated when medical records from one year are used. In general, patients with diabetes or COPD visit their general practitioner frequently throughout a year but patients with osteoarthritis or asthma for example visit their general practitioner infrequently, sometimes even less than once a year. Therefore, only patients registered for a minimum period of 3 consecutive years in a general practice within the LINH network were selected.

### Analyses

The selection of patients registered for a minimum period of 3 consecutive years resulted in a study population of 212,902 patients registered with 59 general practices.

Sex and age standardization was applied in all prevalence estimates to account for differences with the Dutch population in 2008 (http://statline.cbs.nl). The prevalence of chronic diseases and multimorbidity was defined as the number of patients with one or more diseases divided by the total number of 212,902 registered patients. In addition, prevalence estimates were stratified according to sex and age groups. To determine prevalence estimates it was assumed that a chronic disease, once recorded, remains prevalent during all follow-up years in the registration (recovery is not possible).

Analyses for clustering and comorbidity were restricted to 52,014 patients of 55 years and older. If diseases are completely independent of one another, they can be expected to co-occur at a rate which equals the product of the prevalence rates of the separate diseases [5]. For some pairs of diseases, the rate of co-occurrence may be higher than expected, which is referred to as disease clustering [18]. Clustering of chronic diseases is determined by the ratio of the observed prevalence rate and the expected prevalence rate of the pair of diseases. Logistic regression analyses adjusted for sex and age were used to investigate disease clustering.

For each of the 10 chronic diseases the proportion patients without comorbidity and those with 1, 2, 3, and 4 or more comorbid diseases was calculated. Finally, the five most prevalent comorbid chronic diseases (from 28 other diseases) were determined for the 10 specific diseases.

All analyses were performed in SAS version 9.2 (SAS Institute, Cary, North Carolina, USA).

## Results

One-third of all registered patients had at least one chronic disease out of 29 chronic diseases (all ages) and about 13% had multimorbidity (Table2). The prevalence rate of chronic diseases and multimorbidity is higher for women and higher age groups. Of patients of 75 years and older 84% had a chronic disease and 59% had more than one chronic disease. In total, 37% of all patients of 55 years and older was known with multimorbidity.

**Table 2:** Prevalence rates of patients with chronic diseases and patients with multimorbidity according to sex and age, LINH 2002-2008

	**N**	Prevalence rate of patients with chronic diseases (%)*	Prevalence rate of multimorbid patients (%)*
Total	212,902	33.7%	12.9%
Men	105,547	30.7%	10.9%
Women	107,355	36.6%	15.0%
0-14 years	38,944	12.6%	0.6%
15-24 years	26,718	15.2%	2.0%
25-54 years	95,226	30.0%	8.1%
55-64 years	22,592	52.9%	22.7%
65-74 years	17,465	70.0%	39.1%
74+ years	11,957	83.5%	59.2%

* According to the selection of 29 chronic diseases in Table1.

The prevalence estimates for pairs of chronic diseases in the population of 55 years and older are presented in Table3. The prevalence of co-occuring diabetes and coronary heart disease was 3.6% for example, and COPD and osteoarthritis co-occur in 1.7% of the patients. Most odds ratios are significantly higher than 1.0, indicating that almost all pairs of chronic diseases co-occur more frequently than expected on basis of statistical independency of diseases. The highest odds ratios were found for the disease pairs depression and anxiety disorder, coronary heart disease and heart failure, and COPD and heart failure.

**Table 3:** **Prevalence (P) co-occuring chronic diseases (%)**^**1**^**and the sex and age adjusted odds ratio (OR) for clustering for patients of 55 years and older**^**2**^**, LINH 2002–2008**

	**Coronary heart disease**		**Osteoarthritis**		**COPD**		**Chronic neck or back disorder**		**Cancer**		**Stroke**		**Depression**		**Heart failure**		**Anxiety disorder**	
	**P**	**OR**	**P**	**OR**	**P**	**OR**	**P**	**OR**	**P**	**OR**	**P**	**OR**	**P**	**OR**	**P**	**OR**	**P**	**OR**
Diabetes mellitus	3.6	1.9*^3^	2.8	1.1*	2.1	1.3*	2.5	1.3*	1.9	1.1	1.9	1.6*	1.4	1.2*	2.2	1.7*	0.5	1.1
Coronary heart disease			2.5	1.3*	2.2	1.6*	2.3	1.6*	1.8	1.2*	1.5	1.3*	1.3	1.5*	2.8	3.7*	0.4	1.5*
Osteoarthritis					1.7	1.2*	2.8	2.0*	1.7	1.1*	1.2	1.0*	1.4	1.4*	1.6	1.3*	0.4	1.1
COPD							1.5	1.4*	1.4	1.3*	1.0	1.3*	1.0	1.7*	1.9	3.4*	0.4	1.7*
Chronic neck or back disorder									1.4	1.2*	1.0	1.3*	1.2	1.5*	0.9	1.2*	0.5	1.4*
Cancer											0.9	1.0	0.9	1.4*	1.2	1.3*	0.3	1.4*
Stroke													0.8	1.6*	1.2	1.5*	0.2	1.3*
Depression															0.7	1.5*	0.8	5.9*
Heart failure																	0.2	1.5*

^1^Prevalence estimates are standardized according to the age and sex distribution in the Dutch population in 2008.

^2^ The odds ratio represents the ‘probability’ for co-occurrence of 2 chronic diseases. Two chronic diseases co-occur more frequent than would be expected on basis of independency if the odds ratio is larger than 1.0 and the 95% confidence interval does not include 1.0.

^3^ The prevalence of comorbidity of diabetes mellitus and coronary heart disease is 3.6%. Diabetes mellitus and coronary heart disease co-occur significantly more frequent than expected on basis of independency, with an odds ratio of 1.9.

* Co-occurrence of diseases occurred more frequent than expected on basis of independency (p < 0.05).

**Figure 1:**
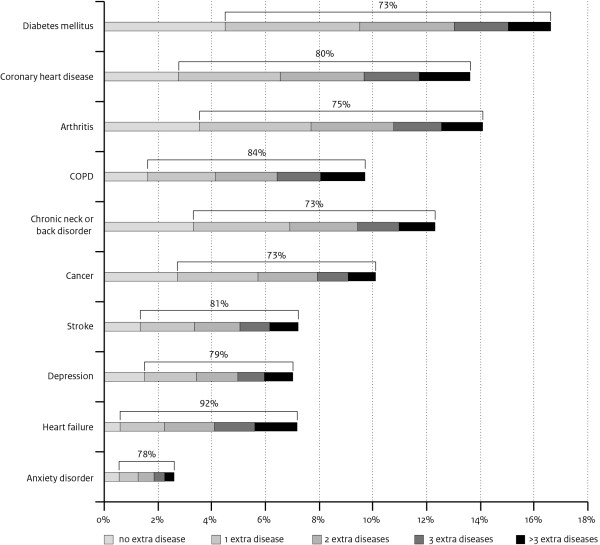
Prevalence of 10 chronic diseases and comorbidity (%) among patients of 55 years and older.

Over 70% of those with one of the selected 10 chronic diseases had also one or more other chronic diseases (comorbidity). The highest proportion of comorbidity is shown for heart failure, of all patients with heart failure 92% had at least one extra chronic disease (Figure1). About a quarter of the patients with heart failure had one extra disease, 23% two extra diseases, 21% three extra diseases, and 22% had four or more extra chronic diseases.

Diabetes mellitus, osteoarthritis, and coronary heart disease were the most prevalent co-occuring diseases for all 10 diseases (Table4). From all patients with cancer and one or more extra diseases, 26% was recorded with diabetes mellitus, 24% with coronary heart disease, 23% with osteoarthritis, 19% with chronic neck- or back disorders, and 18% with COPD. One-third of those with cancer and comorbidity had only diseases included in the top-5 comorbid diseases for cancer, the other patients (two-thirds) had at least one disease that was not included in the top-5 comorbid diseases for cancer. Similar numbers were shown for the 9 other specific diseases (Table4): about 30% of the patients with comorbidity had only diseases included in the top-5 whereas 70% had one or more diseases not included in the top-5.

**Table 4:** Top 5 of most prevalent comorbidity for 10 chronic diseases (first column) and prevalence of co-occuring diseases among all patients of 55 years and older with the specific chronic disease and comorbidity

**Chronic disease**	**Comorbidity top 5 and prevalence**										Contribution of top 5 to all comorbidity with the specific chronic disease*
	**1**		**2**		**3**		**4**		**5**		
**Diabetes mellitus**	Coronary heart disease	30.0%	Osteoarthritis	22.7%	Chronic neck or back disorder	20.3%	Heart failure	18.1%	COPD	17.2%	31.5%
**Coronary heart disease**	Diabetes mellitus	33.5%	Heart failure	25.5%	Osteoarthritis	23.0%	Chronic neck or back disorder	20.8%	Cancer	16.7%	32.8%
**Osteoarthritis**	Chronic neck or back disorder	26.8%	Diabetes mellitus	26.0%	Coronary heart disease	23.6%	Visual disorder	16.4%	Cancer	16.0%	33.5%
**COPD**	Asthma	32.4%	Coronary heart disease	27.4%	Diabetes mellitus	25.8%	Heart failure	24.0%	Osteoarthritis	20.5%	29.9%
**Chronic neck or back disorder**	Osteoarthritis	31.5%	Diabetes mellitus	27.3%	Coronary heart disease	25.2%	COPD	17.0%	Cancer	15.5%	35.8%
**Cancer**	Diabetes mellitus	25.6%	Coronary heart disease	24.4%	Osteoarthritis	22.8%	Chronic neck or back disorder	18.8%	COPD	18.3%	33.1%
**Stroke**	Diabetes mellitus	32.4%	Coronary heart disease	26.0%	Osteoarthritis	21.0%	Heart failure	20.2%	COPD	17.5%	27.8%
**Depression**	Osteoarthritis	24.7%	Diabetes mellitus	24.6%	Coronary heart disease	22.8%	Chronic neck or back disorder	22.3%	COPD	18.0%	26.4%
**Heart failure**	Coronary heart disease	42.0%	Diabetes mellitus	33.3%	COPD	29.4%	Osteoarthritis	24.5%	Stroke	18.0%	30.4%
**Anxiety disorder**	Depression	38.0%	Diabetes mellitus	22.3%	Chronic neck or back disorder	22.2%	Coronary heart disease	20.7%	Osteoarthritis	19.8%	31.4%

^1^ Prevalence is determined by dividing the number of patients with a specific combination of co-occuring diseases through the number of patients with the specific chronic disease and 1 or more other chronic diseases. Prevalence estimates are standardized according to the sex and age distribution in the Dutch population in 2008.

* According to the selection of 29 chronic diseases in Table1.

## Discussion

Our analyses show that multimorbidity is common, especially among older persons, and that among those with a chronic disease over two thirds has other comorbidities. A more detailed look at the combinations of diseases showed a wide variety in multimorbidity. We observed that all chronic diseases tend to cluster, i.e. most disease pairs co-occur more often than expected by chance. Furthermore, the top-5 of co-occurring chronic diseases represents only a minor proportion of the comorbidity: all diseases co-occur together. These findings may have important consequences since the organization and funding of health care is organized by disease-specific programs in many countries. Such disease-specific approaches do not match the reality of most people with a chronic disease.

### Strengths and weaknesses of the study

Primary care registries represent an interesting source to describe multimorbidity of chronic diseases because often most health problems are known and recorded by the general practitioner, especially in countries as the Netherlands where general practice is the entry point for health care and general practitioners act as gatekeeper for secondary care. Analyzing multimorbidity among general practice patients can be used for different research questions [3,4,19,20] but ideally we need to know the underlying population or epidemiological denominator. Strength of general practice registries in the Netherlands is that almost everybody is registered within a general practice. Hence, it is possible to use morbidity data from the general practice registries to describe morbidity of the general population.

In addition, use of long-term registration data of a country-wide network of general practices for the analyses on multimorbidity has several advantages, like the distribution of the general practices over the Netherlands, the relatively long registration period, and the standardized registration procedures. A general strength of general practice medical records compared to self-reported data is the availability of diagnosed chronic diseases. A disadvantage is that diseases for which patients do not consult a general practitioner are not in the general practitioners’ medical record system. In addition, the small group of elderly in nursing homes, usually having more than one chronic disease, are generally not registered within a general practice. Owing to these exclusions the prevalence of chronic diseases in the general population may be underestimated based on general practice registrations. Underdiagnosis and underreporting of health problems like depression may also lead to underestimation of the prevalence of multimorbidity.

Length of follow-up in a registration affects the extent and the reliability of multimorbidity estimates [21]. The frequency of general practitioner consultations for some diseases, such as osteoarthritis, are even less than once a year. The prevalence of those diseases is underestimated in a registration with a one-year follow-up, therefore the minimum patient follow-up in our study was three years. With a longer follow-up period selection may play a role since patients move away and general practices drop out from the registration.

Apparently, the prevalence of multimorbidity in this study is completely determined by the selection of 29 chronic diseases. Generally speaking, the more chronic conditions are included the higher prevalence rates of multimorbidity will be found. We presume that an important part of chronic morbidity is included in our selection of diseases. By counting the number of chronic diseases we did not take into account any differences in the severity of diseases [22]. When interpreting the results of our and similar studies that are based on general practice registrations, it should be noticed that the varying consequences of diseases on patients’ physical and mental functioning, disability and independency are not considered [8,23].

### Comparison with literature

Registry characteristics affect the prevalence and nature of multimorbidity, especially the selection and definition of diseases affect the actual prevalence rates [21,24]. This limits comparisons with other studies, even those based on general practice registries. However, the general patterns we found are similar to those of other studies. First, multimorbidity being rather the rule than the exception and prevalences increasing with age but also found at younger age [3-8,25-27]. Second, clustering of diseases is commonly found in all recent studies on multimorbidity of (chronic) diseases [5,28-32]. Third, the most prevalent diseases like heart disease, diabetes and osteoarthritis end up high in every multimorbidity rank [4,29]. Most studies showed hypertension, obesity and hyperlipidemia to rank high in prevalence when it was defined as a chronic disease [21,28,29]. To our opinion these are chronic *conditions* that increase the risk on chronic disease but are not *diseases*, we therefore did not include these in our analyses. Fourth, a majority of persons with one chronic disease also have at least one (and often more) other disease and these are not limited to a few common diseases, these can be any disease [6,28,29]. While a descriptive approach was used in our analyses and others [6,29] few other studies performed cluster analyses to identify comorbidity patterns [28,30-32]. These studies identified 3 to 6 clusters of diseases and most revealed a cluster with vascular conditions and a cluster with mental diseases along with pain [28,30,32]. We focused on common co-occuring conditions and our findings indicate a wide variety of co-occuring conditions since only 30% of the comorbidity spectrum can be attributed to the 5 most common comorbidities. In line with this, van den Bussche described that combinations of the six most prevalent chronic conditions span only 42% of the comorbidity spectrum [29].

### Implications of the study

A substantial proportion of the older population being characterized by multimorbidity of chronic diseases requires reconsiderations of medical research as well as the organization of care. Most research and clinical practice are based on a single disease paradigm, which may not be appropriate for patients with multiple complex health problems [33,34]. Studies investigating the (cost-)effectiveness of new treatments commonly exclude patients with multimorbidity. Findings from these studies have a very limited reach since multimorbidity affects the majority of the aged. There is thus a clear need to shift research from disease-specific treatments and patients with single diseases to research which takes into account multimorbidity or is explicitly focused on patients with multimorbidity [35].

Multimorbidity leads to complex care through the use of different treatment strategies and the involvement of various health care professionals, which may lead to opposing advices (counseling) or medications [36]. Due to fragmented expertise and focus by different care providers, patients preferences, expectancies, values and needs regarding their daily life are often overlooked [37]. Patients’ main priorities are usually an adequate quality of life and appropriate daily functioning in addition to the improvement of disease-specific health problems.

Currently, disease management programs are implemented worldwide in order to enhance quality and continuity of care for the chronically ill [38,39]. These programs are characterized by a patient-centered approach of coordinated multiple healthcare interventions that structure chronic care to a specific patient group [39,40]. However, participating in multiple single-disease oriented programs in combination with regular primary care, may lead to fragmented care. In designing these programs insufficient attention is paid to multimorbid conditions. Multimorbid patients are therefore at risk for suboptimal treatment, unsafe care, inefficient use of health care services, unnecessary costs and consequently run higher risks for adverse events [41]. Case management is a potential model which might counteract fragmented care for multimorbid patients. It is an individualized care program which coordinates all care involved for patients enrolled in different single-disease management programs, who have to adhere to various treatment protocols. It draws on evidence-based optimal care for systematically managing all existing conditions in a patient, and is tailored to the individual patients’ preferences [42-44].

The finding that multimorbidity cannot be captured with a few common combinations of diseases represents a challenge for disease-specific treatment as well as disease-specific disease management programs and rises the question for which patient group case management should be implemented. Given the enormous variety in multimorbidity, we need more knowledge about the co-occurring conditions that are leading to a high need for care, a major decline in quality of life, and/or an increase in functional limitations.

## Conclusion

Multimorbidity is common at all ages and cannot be captured by a few common combinations of diseases.

## Competing interests

The authors declare that they have no competing interests.

## Authors’ contributions

SO, HP, LL, BG, NH, and CB participated in the conceptual development, study design and the interpretation of the results. SO and HP drafted the manuscript and SO analysed the data. LL, BG, NH, CD, RV, FG, and CB revised the manuscript. RV, FG and CD participated in data collection. All authors read and approved the final manuscript.

## The submitted manuscript is an adapted and extended version of a Dutch paper

van Oostrom SH, Picavet HS, van Gelder BM, Lemmens LC, Hoeymans N, Verheij RA, Schellevis FG, Baan CA: Multimorbiditeit en comorbiditeit in de Nederlandse bevolking – gegevens van huisartsenpraktijken [Multimorbidity and comorbidity in the Dutch population - data from general practices]. *Ned Tijdschr Geneeskd* 2011, 155:A3193.

## Author details

Author details^1^Centre for Prevention and Health Services Research, National Institute for Public Health and the Environment, P.O. Box 1, Bilthoven 3720 BA, the Netherlands. ^2^Centre for Public Health Forecasting, National Institute for Public Health and the Environment, P.O. Box 1, Bilthoven 3720 BA, the Netherlands. ^3^Department of General Practice, Netherlands Institute for Health Services Research, P.O. Box 1568, Utrecht 3500 BN, the Netherlands.^4^Department of General Practice/EMGO Institute, VU University Medical Centre, Amsterdam, the Netherlands.

## Pre-publication history

The pre-publication history for this paper can be accessed here:

http://www.biomedcentral.com/1471-2458/12/715/prepub
